# Protein profiling of testicular tissue from boars with different levels of hyperactive sperm motility

**DOI:** 10.1186/s13028-022-00642-1

**Published:** 2022-09-05

**Authors:** Maren van Son, Dag Inge Våge, Morten Skaugen, Nina Hårdnes Tremoen, Ann Helen Gaustad, Teklu Tewoldebrhan Zeremichael, Frøydis Deinboll Myromslien, Eli Grindflek

**Affiliations:** 1 Norsvin, Storhamargata 44, 2317 Hamar, Norway; 2 Department of Animal and Aquacultural Sciences, Faculty of Biosciences, Centre for Integrative Genetics (CIGENE), Norwegian University of Life Sciences, 1432 Ås, Norway; 3 Faculty of Chemistry, Biotechnology and Food Sciences, Norwegian University of Life Sciences, 1432 Ås, Norway; 4 Department of Biotechnology, Inland Norway University of Applied Sciences, 2318 Hamar, Norway

**Keywords:** Pig, Proteome, Sperm hyperactivity, Sperm motility

## Abstract

Hyperactive sperm motility is important for successful fertilization. In the present study, a proteome profiling approach was performed to identify the differences between Landrace boars with different levels of hyperactive sperm motility in liquid extended semen. Two contrasts were studied: (i) high versus low levels of sperm hyperactivity at semen collection day and (ii) high versus low change in levels of sperm hyperactivity after 96 h semen storage. Testicular samples were analyzed on a Q Exactive mass spectrometer and more than 6000 proteins were identified in the 13 samples. The most significant differentially expressed proteins were mediator complex subunit 28 (MED28), cell division cycle 37 like 1 (CDC37L1), ubiquitin specific peptidase 10 (USP10), zinc finger FYVE-type containing 26 (ZFYVE26), protein kinase C delta (PRKCD), actinin alpha 4 (ACTN4), *N*(alpha)-acetyltransferase 30 (NAA30), C1q domain-containing (LOC110258309) and uncharacterized LOC100512926. Of the differentially expressed proteins, 11 have previously been identified as differentially expressed at the corresponding mRNA transcript level using the same samples and contrasts. These include sphingosine kinase 1 isoform 2 (SPHK1), serine and arginine rich splicing factor 1 (SRSF1), and tubulin gamma-1 (TUBG1) which are involved in the acrosome reaction and sperm motility. A mass spectrometry approach was applied to investigate the protein profiles of boars with different levels of hyperactive sperm motility. This study identified several proteins previously shown to be involved in sperm motility and quality, but also proteins with no known function for sperm motility. Candidates that are differentially expressed on both mRNA and protein levels are especially relevant as biological markers of semen quality.

**Supplementary Information:**

The online version contains supplementary material available at 10.1186/s13028-022-00642-1.

## Findings

The quality of semen in terms of fertilization rate is critical for efficient use of artificial insemination, and parameters that can predict the fertilization rate of semen samples are valuable. One important parameter is the level of hyperactive sperm motility, which is a movement pattern of spermatozoa after capacitation, normally taking place in the oviduct. The increased vigor in swimming movement makes the sperm more efficient in ascending the oviduct and penetrating the zona pellucida [1]. Computer-Assisted Semen Analysis (CASA) is an objective examination of semen characteristics [2] and we have previously shown that several CASA parameters defining hyperactive sperm motility was correlated to total number of piglets born [3]. Moreover, the Landrace pig breed developed a more hyperactive swimming pattern during storage compared to the Duroc breed, which is relevant as semen is usually stored before insemination. However, if hyperactive sperm motility is acquired too early, sperm cells may deplete their energy and die before reaching oocytes.

Genetic factors related to fertility are interesting as they can be used to ensure good semen quality. We have previously identified differentially expressed (DE) genes in testis related to levels of hyperactive sperm motility [4]. The aim of the current study was to gain further insights into the molecular background causing sperm hyperactivity by performing protein profiling of the same testicular samples as previously used in mRNA expression. This allowed us to establish differentially expressed proteins related to hyperactive sperm motility as well as investigate the correlation of the proteome to the transcriptome.

The animals, ejaculate samples, testicular tissue samples and CASA analysis used in this study have been described [4]. Data are also available in Additional file 1. From the CASA analysis, hyperactive motility for each single sperm cell track were defined as VCL > 97 µm/s, ALH > 3.5 µm, LIN < 32% and WOB < 71%. Testicular tissue was collected after slaughter and immediately frozen in liquid nitrogen and stored at -80 °C until protein extraction. Protein extraction was done using the Qproteome Mammalian Protein Prep kit [Qiagen, Germany] and the protocol “Purification of protein from animal tissues using the Qproteome Mammalian Protein Prep kit and the TissueRuptor”. Methods used for in-gel digestion, peptide clean-up and liquid chromatography-mass spectrometry are also described in detail in Additional file 1. Peak lists were generated from raw data files by the MaxQuant software and proteins were identified from the Uniprot porcine reference protein database. We performed protein DE analyses using the Bioconductor Differential Enrichment analysis of Proteomics data (DEP) package [5] and the Perseus software [6]. The DEP workflow started from MaxLFQ values obtained from MaxQuant, which are intensity values for each protein in each sample. Filtering removed proteins that were potential contaminants or originated from reverse sequences. Only proteins that were missing in maximum one sample were kept and normalization was done using the Variance Stabilizing Normalization approach [7]. Missing values were present in some samples due to low intensities, and these were imputed using random draws from a Gaussian distribution centered around a minimal value (option “MinProb”). Correction for multiple testing was done by the R package fdrtool [8] and DE was considered significant with an FDR of 0.05 and a log2 fold change of 0.6 (corresponding to a fold change of 1.5 on a linear scale). A workflow in Perseus v.1.6.7 was created starting from the same MaxLFQ values. Proteins that were only identified by a modification site, potential contaminants, or reverse sequences were removed. The dataset was log2 transformed and filtered so that each protein was present in at least 70% of the samples. Two-sample t-tests were performed for both contrasts. A p-value of 0.05 with log2 fold change of 0.6 was applied to indicate significance.

Two different contrasts were examined in this study: (i) high versus low sperm hyperactivity at collection and (ii) high versus low change in levels of hyperactivity after 96 h storage (Table 1). Samples L2 and L11 are from the same boar and have extreme values in both contrasts, the same is the case for samples L7 and L14. Sample L2/L11 clustered as an outlier in both contrasts by hierarchical clustering and was excluded from DE analyses. The mean (± SD) hyperactivity values for the subsequent high and low groups were 14.5% (± 1.7) (n = 4) and 1.7% (± 0.2) (n = 3) for contrast (i) and 15.1% (± 3.0) (n = 3) and 2.8% (± 0.6) (n = 4) for contrast (ii). The data analysis obtained 66,366 unique peptides, which were assembled into 6,362 proteins (see Additional file 2).

**Table 1:** Sperm hyperactivity measurements for the different boars included in this study

Group	Boar	Number of ejaculates	Mean %hyperactivity	SD %hyperactivity
Landrace low at collection	L1	3	1.9	0.2
	L2	4	1.2	0.7
	L3	3	1.6	0.6
	L4	3	1.6	0.2
Landrace high at collection	L5	3	16.4	7.3
	L6	3	13.6	3.2
	L7	4	15.5	3.1
	L8	4	12.6	3.1
Landrace low change after 96 h	L9	5	3.3	2.1
	L10	3	2.1	1.4
	L11	4	2.3	0.6
	L12	3	3.0	2.8
Landrace high change after 96 h	L13	3	11.7	7.0
	L14	4	17.3	9.0
	L15	3	16.2	3.7

The boars of contrast (i) (L1–L8) and (ii) (L9–L15) are presented with the number of ejaculates, mean and standard deviation (SD) for % sperm hyperactivity in the ejaculates

Using the DEP analysis, 32 and 16 proteins were found DE for contrasts (i) (see Additional file 3: Table X1) and (ii) (see Additional file 4: Table X1), respectively. The most significant DE proteins for contrast (i) were mediator complex (MED28), cell division cycle 37 like (CDC37L1), zinc finger FYVE-type containing 26 (ZFYVE26), ubiquitin carboxyl-terminal hydrolase (USP10) and uncharacterized LOC100512926 (all with *q*-value 1.76e−13). For contrast (ii), the most significant DE proteins were *N*(alpha)-acetyltransferase 30 (NAA30) and C1q domain-containing (LOC110258309) (both with *q*-value of 9.46e−13).

Using the Perseus analysis procedure, 44 proteins were DE in contrast (i) (see Additional file 3: Table X2) and 87 proteins in contrast (ii) (see Additional file 4: Table X2). The most significant DE protein of contrast (i) was protein kinase C (PRKCB) (P-value = 0.0004), whereas for contrast (ii), the most significant protein was actinin alpha 4 (ACTN4; P-value = 0.0003).

Results obtained by both DEP and Perseus workflows showed that nine and five proteins were significantly DE for contrast (i) and (ii), respectively, using both analysis methods (Table 2). Comparing results for the two different contrasts indicated that 10 proteins are relevant for sperm hyperactivity both at collection and after 96 h storage (Table 3). Of these, PRKCB, parvalbumin alpha (PVALB), sphingosine kinase 1 (SPHK1) (Table 2), ACTN4 and tubulin alpha chain (TUBA8) (Tables 2 and 3), have previously been associated with sperm quality. PRKCB is a protein kinase involved in sperm motility and acrosome reaction [9]. The *ACTN4* gene was previously found up-regulated in Landrace boars with high sperm DNA fragmentation index [10], where the boars were from the same population as the current study. PVALB has previously been associated with sperm motility in carp [11] whereas TUBA8 levels were associated with reduced sperm motility in human [12]. SPHK1 was implicated to be involved in the acrosome reaction in mice [13].

**Table 2:** Differentially expressed proteins found in both analysis methods

Contrast	UniProt ID	Protein name	*q*-value	P-value	FC D	FC P	Gene name
i	K9J4L8	Protein kinase C	0.03	0.0004	− 1.01	− 1.26	*PRKCB*
i	F1SJ00	LisH domain-containing protein	0.03	0.0005	− 1.97	− 2.5	*DCAF1*
i	A0A286ZNB8	Lysophospholipase-like protein 1 isoform a	0.005	0.01	0.87	0.89	*LYPLAL1*
i	F1SR79	J domain-containing protein	0.03	0.01	− 0.78	− 0.81	*DNAJB2*
i	F1RWP6	Sphingosine kinase 1 isoform 2	0.01	0.02	0.86	0.78	*SPHK1*
i	F1SDG0	Uncharacterized protein	6.4e−05	0.02	− 1.37	− 1.12	*SF3B4*
i	F1SNT1	Uncharacterized protein	0.04	0.02	− 0.69	− 0.66	*ACAD11*
i	F1S5S7	Inactive hydroxysteroid dehydrogenase-like protein 1 isoform X1	0.04	0.03	− 1.06	− 1.06	*HSDL1*
i	F1SKJ8	Parvalbumin alpha	0.03	0.03	− 1.62	− 0.95	*PVALB*
ii	A0A287BAZ5	Uncharacterized protein	0.05	0.0003	− 0.7	− 0.74	*ACTN4*
ii	I3LFS1	Uncharacterized protein	0.02	0.004	− 0.9	− 0.87	*USP39*
ii	F2Z5T8	Uncharacterized protein	7.0e−08	0.007	− 2.72	− 2.38	*MOBKL3*
ii	A0A287B5H5	Tubulin alpha chain	0.03	0.009	− 0.94	− 0.99	*TUBA8*
ii	F1SFI7	Alpha-2-HS-glycoprotein	0.05	0.01	1.01	0.93	*AHSG*

Common proteins using the two analysis methods for contrast (i) and (ii) with UniProt ID, protein name, *q*-value for DEP, p-value for Perseus, fold change (FC) for DEP (D) and Perseus (P) and corresponding gene name

**Table 3:** Differentially expressed proteins found for both contrast (i) and (ii)

Method	UniProt ID	Protein name	Gene name
DEP	A0A287BAZ5	Uncharacterized protein	*ACTN4*
	I3LFS1	Uncharacterized protein	*USP39*
	F2Z5T8	Uncharacterized protein	*MOBKL3*
	A0A287B5H5	Tubulin alpha chain	*TUBA8*
	F1SFI7	Alpha-2-HS-glycoprotein	*AHSG*
Perseus	K7GND3	ATP-dependent RNA helicase A	*DHX9*
	A0A287BE52	LEDGF domain-containing protein	*PSIP1*
	F1S5S7	Inactive hydroxysteroid dehydrogenase-like protein 1 isoform X1	*HSDL1*
	A0A287AIE8	Uncharacterized protein	*SUGP2*
	I3LDD5	Pribosyltran domain-containing protein	*PRTFDC1*

Common proteins found for contrast (i) and (ii) using DEP or Perseus, presented with UniProt ID, Protein name and corresponding gene name

By comparing results of the proteome profiling to those previously obtained in transcriptome profiling of the same samples [4], 10 were in common for contrast (i) and one for contrast (ii) (Table 4). Some of these candidates have previously been shown to have a role in spermatogenesis and sperm motility, supporting a role also in hyperactive motility. Knock-out of the serine/arginine-rich splicing factor 1 (SRSF1) in mice showed that this protein is essential for sperm motility [14]. Tubulin gamma 1 (TUBG1) is suggested to have an important role in sperm motility in human samples [15]. Solute carrier family 12A7 (SLC12A7) is a NaCl cotransporter [16], making it an interesting candidate for sperm hyperactivity as Na ions are necessary for sperm motility, whereas WD repeat domain 13 (WDR13) is regulated by genes essential to normal spermatogenesis [17]. SPHK1 is already described above as it is one of the proteins identified by both analysis methods. The exact function of the other proteins listed in Table 4 needs to be further investigated, however based on their differential expression both on mRNA and protein level, we suggest an important role in hyperactive sperm motility.

**Table 4:** Complementary protein and transcriptome results for high versus low hyperactivity at collection (i) and after 96 h storage (ii)

Gene symbol	Protein name	UniProt ID	Method	Contrast
SLC12A7	Uncharacterized protein	I3L7H3	DEP	i
SPHK1	Sphingosine kinase 1 isoform 2	F1RWP6	DEP, Perseus	i
WDR13	Uncharacterized protein	I3LPL0	DEP	i
NUCKS1	Uncharacterized protein	A0A287AWH5	DEP	i
CDC37L1	Hsp90 co-chaperone Cdc37-like 1	F1SK44	DEP	i
FAM98C	Protein FAM98C	F1RI46	Perseus	i
OSBPL2	Oxysterol-binding protein	A0A286ZQP4	Perseus	i
MSMO1	Methylsterol monooxygenase 1	D0G7F0	Perseus	i
TUBG1	Tubulin gamma chain	F2Z562	Perseus	i
SRSF1	Serine/arginine-rich splicing factor 1	Q3YLA6	Perseus	i
CREG1	Uncharacterized protein	F1S268	Perseus	ii

The correlating protein and transcriptome expression data is presented with gene symbol, protein name, UniProt ID, analysis method used to detect significance on protein level and contrast where differential expression was detected

In conclusion, we identified DE proteins in testicular samples from pigs related to levels of hyperactive sperm motility. Eleven candidates were significant both on protein and mRNA level and are highly relevant as potential biomarkers for pig fertility.

## Supplementary Information

**Additional file 1.** Methods used for in-gel digestion, peptide clean-up and liquid chromatography-mass spectrometry.

**Additional file 2.** Peptides and proteins identified in the samples of this study. The data analysis revealed that 66,366 unique peptides (Table X1), which were assembled into 6362 proteins (Table X2), were identified.

**Additional file 3.** Differentially expressed proteins for contrast (i) high versus low hyperactivity at collection. Results presented as analyzed by Perseus (Table X1) and as analyzed by DEP (Table X2) presented with UniProt ID, protein name, *q*-value, log2 fold change and corresponding gene name.

**Additional file 4.** Differentially expressed proteins for contrast (ii) high versus low change in levels of hyperactivity after 96 h storage. Results as analyzed by Perseus (Table X1) and as analyzed by DEP (Table X2) presented with UniProt ID, protein name, *q*-value, log2 fold change and corresponding gene name.

## Data Availability

The mass spectrometry proteomics data have been deposited to the ProteomeXchange Consortium via the PRIDE partner repository with the dataset identifier PDX028528.

## Abbreviations

ABC: Ammonium bicarbonate
ACN: Acetonitrile
ACTN4: Actinin alpha 4
CASA: Computer-Assisted Semen Analysis
CDC37L1: Cell division cycle 37 like 1
DE: Differential expression/differentially expressed
DEP: Differential Enrichment analysis of Proteomics data
MED28: Mediator complex MED28
NAA30: *N*(alpha)-Acetyltransferase 30, NatC catalytic subunit
PRKCB: Protein kinase C
PVALB: Parvalbumin alpha
SLC12A7: Solute carrier family 12A7
SPHK1: Sphingosine kinase 1
SRSF1: Serine/arginine-rich splicing factor 1
TUBA8: Tubulin alpha chain
TUBG1: Tubulin gamma-1
USP10: Ubiquitin carboxyl-terminal hydrolase
WDR13: WD repeat domain-13
ZFYVE26: Zinc finger FYVE-type containing 26
