# Supplementary material for: Protein profiling of testicular tissue from boars with different levels of hyperactive sperm motility
Source: Acta Vet Scand. 2022 Sep 5;64:21. doi: 10.1186/s13028-022-00642-1 (PMC9446748; doi:10.1186/s13028-022-00642-1)
Supplement: Supplementary file 1 — Additional file 1. Methods used for in-gel digestion, peptide clean-up and liquid chromatography-mass spectrometry. [file 13028_2022_642_MOESM1_ESM.docx]

**Materials**

Semen samples from 239 ejaculates of 103 different purebred Norwegian Landrace boars were collected at the AI station run by Norsvin at Hamar, Norway. All boars included in this study were routinely used for AI and the ejaculates were part of routine collections.

After slaughter, a piece of testicular tissue was cut with a scalpel from the middle of the testicle. Testicular tissue samples were frozen immediately in liquid nitrogen before storage at -80°C until protein extraction.

**CASA measurements**

At the AI station, motility and morphology were evaluated using phase contrast microscopy [Leica DM 4000B, Leica Microsystems, Germany] at 37°C, and ejaculates with <70% motile and/or >20% morphologically abnormal spermatozoa were discarded. Approved ejaculates were diluted to a final concentration of 25 x 10^6^ cells/mL in Androstar® Plus extender [Minitube, Germany], transferred to airtight tubes containing doses of 89 mL and stored at 18°C until shipment. At the laboratory, the samples were transferred to 15 mL falcon tubes and analyzed by CASA at the day of collection and after 96 hours storage at 18°C. In order to be included in the selection of extreme animals in this study, the boars needed at least three consistent measurements on % hyperactivity. Sperm motility analysis was conducted using Sperm Vision CASA system [SpermVision, Germany] with Leja-4 standardized counting chambers [Leja products, the Netherlands] and analyzed using a phase contrast microscope [Axio Lab.A1, Germany]. The Sperm Vision and the Leja-4 slides were pre-warmed at 38°C and the boar ejaculate samples were incubated at 38°C for 10 minutes prior to CASA analysis. The capillary flow chambers of the Leja counting slides were filled with 3 µL of pre-warmed semen. Analysis was conducted on eight microscope fields with at least 500 cells analyzed per sample. The mean of eight fields were used for analysis. The criteria for hyperactive motility for each single sperm cell track were VCL>97 µm/s, ALH>3.5 µm, LIN <32% and WOB <71%.

**In-gel digestion and peptide clean-up**

Approximately 50 µg of each protein extract was denatured and loaded on 10% NuPAGE polyacrylamide gels. Electrophoresis was carried out at 200 V for 15-20 minutes, allowing proteins to run 2-3 centimeters into the gel. After Coomasssie staining and de-staining, the gel was transferred to a clean sheet of Al foil and divided into 5 fractions using a clean scalpel blade. After trimming away unstained gel, the gel fractions were further divided into 1-2 mm cubes and transferred to clean 0.2 ml PCR tubes. 100 µl of 50 % acetonitrile (ACN), 50 mM ammonium bicarbonate (ABC) was added to each tube, which were then incubated at rt with shaking for 10 minutes. After a brief centrifugation, the liquid was aspirated and replaced with 200 µl 100 % ACN. The tubes were incubated (rt, shaking) for 15 minutes and the liquid removed by aspiration.

In-gel reduction was performed by adding 50 µl 10 mM DTT, 50 mM ABC to the dried gel pieces, and incubating for 30 minutes at 56 °C in a thermocycler. Alkylation was performed by replacing the solution with 50 µl of 50 mM iodoacetamide, 50 mM ABC, and incubating in the dark for 20 min at rt.

After having removed the alkylation solution, 200 µl of 100% ACN was added and the tubes were incubated (rt, shaking) for 15 minutes, followed by liquid removal and brief air drying of the gel pieces. The tubes were put on ice, and 30 µl ice-cold trypsin solution (13 ng/µl, in 50 mM ABC) was added to each tube. The gel pieces were allowed to swell for a total of 90 minutes on ice, with occasional checks to ensure that they were completely covered with the digestion solution. Finally, the tubes were transferred to a thermocycler and incubated overnight at 37 °C.

Trypsin digestion was terminated by adding 50 µl TFA solution (final concentration 0.2%), and the tubes were sonicated for 10 min in a water bath sonicator. After a brief centrifugation the liquid was transferred to a clean tube. 50 µl of 0.1% TFA was added to the gel pieces and the sonication step was repeated. After combining the two extracts, peptides were purified using STAGE spin-tips, essentially as described by [1]. Eluted peptides were dried in an Eppendorf Concentrator Plus vacuum centrifuge, and dissolved in loading solution (0.05% TFA, 2% ACN in milliQ water) before LC-MS/MS analysis.

**Liquid chromatography – mass spectrometry**

Samples were loaded onto a trap column (Acclaim PepMap100, C_18_, 5 µm, 100 Å, 300 µm i.d. x 5 mm, Thermo Scientific) and backflushed onto a 50 cm analytical column (Acclaim PepMap RSLC C_18_, 2 µm, 100 Å, 75 µm i.d., Thermo Scientific). Starting conditions were 96 % solution A [0.1 % (v/v) formic acid], 4% solution B [80 % (v/v) ACN, 0.1 % (v/v) formic acid]. Peptides were eluted using a flow rate of 300 nl/min using a 70 min method, with the following gradient: from 3.2 to 10 % B in 3 minutes, 10 to 35 % B in 94 minutes and 35 to 60% B in 3 minutes, followed by a 5 min wash at 80 % B and a 15 min equilibration at 4% B. The Q-Exactive mass spectrometer was operated in data-dependent acquisition (DDA) mode using a Top10 DDA method, where acquisition alternates between orbitrap-MS and higher-energy collisional dissociation (HCD) orbitrap-MS/MS acquisition of the 10 most intense precursor ions. Only charge states 2-5 were selected for fragmentation, and the normalized collision energy (NCE) was set to 28. The selected precursor ions were excluded for repeated fragmentation for 20 seconds. The resolution was set to R=70,000 and R=17,500 for MS and MS/MS, respectively. Automatic gain control values were set to 3x10^6^ and 5x10^4^ for MS and MSMS, respectively, with a maximum injection time of 100 and 128 ms.

1. Yu Y, Smith M, Pieper R: **A spinnable and automatable StageTip for high throughput peptide desalting and proteomics**. *Protocol Exchange* 2014.
